# Increased body mass index is associated with improved overall survival in extranodal natural killer/T-cell lymphoma, nasal type

**DOI:** 10.18632/oncotarget.13988

**Published:** 2016-12-16

**Authors:** Ya-Jun Li, Ping-Yong Yi, Ji-Wei Li, Xian-Ling Liu, Xi-Yu Liu, Fang Zhou, Zhou OuYang, Zhong-Yi Sun, Li-Jun Huang, Jun-Qiao He, Yuan Yao, Zhou Fan, Tian Tang, Wen-Qi Jiang

**Affiliations:** ^1^ Department of Lymphoma and Hematology, Hunan Cancer Hospital, Changsha, Hunan, China; ^2^ The Affiliated Cancer Hospital of Xiangya School of Medicine, Central South University, Changsha, Hunan, China; ^3^ Cancer Center of the Second Xiangya Hospital, Central South University, Changsha, Hunan, China; ^4^ Radioactive Interventional Department, Hunan Cancer Hospital, Changsha, Hunan, China; ^5^ State Key Laboratory of Oncology in South China, Guangzhou, China; ^6^ Department of Medical Oncology, Sun Yat-Sen University Cancer Center, Guangzhou, China

**Keywords:** body mass index, extranodal natural killer/T-cell lymphoma, prognosis, IPI, KPI

## Abstract

Objectives: The role of body mass index (BMI) in lymphoma survival outcomes is controversial. The prognostic significance of BMI in extranodal natural killer (NK)/T-cell lymphoma (ENKTL) is unclear. We evaluated the prognostic role of BMI in patients with ENKTL.

Methods: We retrospectively analyzed 742 patients with newly diagnosed ENKTL. The prognostic value of BMI was compared between patients with low BMIs (< 20.0 kg/m^2^) and patients with high BMIs (≥ 20.0 kg/m^2^). The prognostic value of the International Prognostic Index (IPI) and the Korean Prognostic Index (KPI) was also evaluated and compared with that of the BMI classification.

Results: Patients with low BMIs tended to exhibit higher Eastern Cooperative Oncology Group performance status (ECOG PS) scores (≥ 2) (P = 0.001), more frequent B symptoms (P < 0.001), lower albumin levels (P < 0.001), higher KPI scores (P = 0.03), and lower rates of complete remission (P < 0.001) than patients with high BMIs, as well as inferior progression-free survival (PFS, P = 0.003), and inferior overall survival (OS, P = 0.001). Multivariate analysis demonstrated that age > 60 years, mass > 5 cm, stage III/IV, elevated LDH levels, albumin levels < 35 g/L and low BMIs were independent adverse predictors of OS. The BMI classification was found to be superior to the IPI with respect to predicting patient outcomes among low-risk patients and the KPI with respect to distinguishing between intermediate-low- and high-intermediate-risk patients.

Conclusions: Higher BMI at the time of diagnosis is associated with improved overall survival in ENKTL. Using the BMI classification may improve the IPI and KPI prognostic models.

## INTRODUCTION

Despite the pathogenic role of obesity in the development of cancer, the impact of body mass index (BMI) on survival is controversial. In breast cancer and colon cancer, increased BMI were reportedly associated with an increased risk of disease recurrence and death [1, 2]. In contrast, increased BMI were associated with improved survival in patients with lung cancer, gastric cancer and nasopharyngeal carcinoma [3–5]. Previous studies evaluating the impact of BMI on survival in lymphoma have yielded controversial results. Several recent studies found that increased BMI was associated with improved overall survival in patients with non-Hodgkin's lymphoma (NHL) and Hodgkin's lymphoma (HL) [6–10]. However, two studies involving patients with NHL found that increased BMI were associated with decreased survival [11, 12], and another found that BMI was not significantly associated with clinical outcomes among patients with diffuse large B-cell lymphoma (DLBCL), HL or follicular lymphoma (FL) [13].

Extranodal natural killer (NK)/T-cell lymphoma (ENKTL), nasal type, is very rare in Western countries but is relatively common in Asia [14, 15]. The treatment outcomes of ENKTL are generally poor with 5-year overall survival (OS) rates of less than 50% [14, 16–18]. Although the prognostic value of the International Prognostic Index (IPI) has been well validated in many subtypes of NHL, its prognostic value in ENKTL remains controversial [14, 19, 20]. The prognostic significance of the Korean Prognostic Index (KPI) in ENKTL was recently confirmed by several studies. This model may be improved using other laboratory and clinical parameters (e.g., C-reactive protein, albumin, and BMI) [14, 17, 21].

Although the prognostic role of BMI in lymphomas has been investigated in several studies, most of these studies focused on B-cell NHL subtypes. To the best of our knowledge, the prognostic value of BMI in mature T/NK-cell NHL, particularly ENKTL, has never been investigated. We therefore performed this triple-center study to evaluate the prognostic significance of BMI in patients with ENKTL.

## RESULTS

### Patient characteristics

A total of 742 patients (524 male, 218 female; median age, 43 years, range: 10-82 years) met the inclusion criteria. The clinical characteristics of these 742 patients are listed in Table 1. Most patients (724 cases, 97.6%) exhibited favorable performance statuses (ECOG PS scores 0-1). Three hundred and fifty patients (47.2%) presented with B symptoms. A total of 211 patients (28.4%) presented with elevated LDH levels. Seventy patients (9.4%) had masses ≥ 5 cm, and only 16 patients (2.2%) displayed bone marrow involvement. A total of 182 patients (24.5%) exhibited regional lymph node involvement, and 85 patients (11.5%) exhibited more than 2 sites of extranodal involvement. Most patients (654 cases, 88.1%) had localized disease (stage I/II). According to the IPI, the majority of patients (694 cases, 93.5%) were classified as low/low-intermediate risk (IPI = 0-2), while 48 patients (6.5%) were categorized as intermediate-high/high risk (IPI = 3-5). The number of patients with a KPI = 0-1 (500 cases, 67.4%) was significantly higher than the number of patients with a KPI = 2-4 (242 cases, 32.6%). A total of 173 patients (23.3%) presented with hypoalbuminemia ( < 35 g/L). Baseline CRP levels were available for 240 patients (range: 0.16-154.92 mg/L, median value: 7.0 mg/L), and baseline plasma EBV-DNA data were available for 177 patients (range: 0-48,500,000 copies/ml, median value: 1,530 copies/ml).

**Table 1:** Basic clinical characteristics stratified by BMI (body mass index)

Characteristics	BMI groups				P	BMI groups		*P*
	Underweight (< 18.5)	Normal (18.5–24.9)	Overweight (25–29.9)	Obese (≥ 30)		Low BMI (< 20)	High BMI (≥ 20)	
Age (years)					0.878			0.468
median (range)	36 (10–76)	45 (13–82)	44 (20–76)	40 (25–72)		40 (10–79)	45 (15–82)	
≤60	116 (87.2)	441 (85.1)	75 (87.2)	4 (80.0)		221 (87.0)	415 (85.0)	
>60	17 (12.8)	77 (14.9)	11 (12.8)	1 (20.0)		33 (13.0)	73 (15.0)	
Gender					0.219			0.279
male	85 (63.9)	371 (71.6)	65 (75.6)	3 (60.0)		173 (68.1)	351 (71.9)	
female	48 (36.1)	147 (28.4)	21 (24.4)	2 (40.0)		81 (31.9)	137 (28.1)	
ECOG PS					0.018			0.001
0–1	125 (94.0)	508 (98.1)	86 (100)	5 (100)		241 (94.9)	483 (99.0)	
≥2	8 (6.0)	10 (1.9)	0 (0)	0 (0)		13 (5.1)	5 (1.0)	
B symptoms					<0.001			<0.001
Yes	86 (64.7)	237 (45.8)	25 (29.1)	2 (40.0)		153 (60.2)	197 (40.4)	
No	47 (35.3)	281 (54.2)	61 (70.9)	3 (60.0)		101 (39.8)	291 (59.6)	
LDH >245 U/l					0.277			0.094
Yes	39 (29.3)	153 (29.5)	17 (19.8)	2 (40.0)		82 (32.3)	129 (26.4)	
No	94 (70.7)	365 (70.5)	69 (80.2)	3 (60.0)		172 (67.7)	359 (73.6)	
Mass ≥5 cm					0.399			0.590
Yes	17 (12.8)	47 (9.1)	6 (7.0)	0 (0)		26 (10.2)	44 (9.0)	
No	116 (87.2)	471 (90.9)	80 (93.0)	5 (100)		228 (89.8)	444 (91.0)	
Extranodal sites ≥2					0.452			0.094
yes	19 (14.3)	59 (11.4)	7 (8.1)	0 (0)		36 (14.2)	49 (10.0)	
No	114 (85.7)	459 (88.6)	79 (91.9)	5 (100)		218 (85.8)	439 (90.0)	
Regional LN involvement					0.341			0.398
Yes	40 (30.1)	118 (22.8)	23 (26.7)	1 (20.0)		67 (26.4)	115 (23.6)	
No	93 (69.9)	400 (77.2)	63 (73.3)	4 (80.0)		187 (73.6)	373 (76.4)	
Albumin (g/L)					<0.001			<0.001
< 35	46 (34.6)	118 (22.8)	8 (9.3)	1 (20.0)		84 (33.1)	89 (18.2)	
≥ 35	87 (65.4)	400 (77.2)	78 (90.7)	4 (80.0)		170 (66.9)	399 (81.8)	
EBV-DNA^a^ (copies/ml)					0.523			0.158
<1,530	9 (36.0)	65 (51.6)	13 (54.2)	1 (50.0)		23 (41.8)	65 (53.3)	
≥1,530	16 (64.0)	61 (48.4)	11 (45.8)	1 (50.0)		32 (58.2))	57 (46.7)	
CRP ^b^ (mg/L)					0.092			0.061
≤ 10	16 (42.1)	107 (59.8)	15 (68.2)	0 (0)		42 (49.4)	96 (61.9))	
> 10	22 (57.9)	72 (40.2)	7 (31.8)	1 (100)		43 (50.6)	59 (38.1)	
Ann Arbor stage					0.841			0.491
I/II	116 (87.2)	456 (88.0)	77 (89.5)	5 (100)		221 (87.0)	433 (88.7)	
III/IV	17 (12.8)	62 (12.0)	9 (10.5)	0 (0)		33 (13.0)	55 (11.3)	
IPI score					0.746			0.110
0–1	113 (85.0)	439 (84.7)	75 (87.2)	5 (100)		209 (82.3)	423 (86.7)	
2–5	20 (15.0)	79 (15.3)	11 (12.8)	0 (0)		45 (17.7)	65 (13.3)	
KPI score					0.149			0.03
0–1	81 (60.9)	355 (68.5)	59 (68.6)	5 (100)		158 (62.2)	342 (70.1)	
2–4	52 (39.1)	163 (31.5)	27 (31.4)	0 (0)		96 (37.8)	146 (29.9)	

Abbreviations: ECOG PS: Eastern Cooperative Oncology Group performance status; LDH: lactate dehydrogenase; LN: lymph node; EBV-DNA: Epstein–Barr virus-DNA; CRP: C-reactive protein; IPI: International Prognostic Index; KPI: Korean Prognostic Index.

^a^ Data of EBV-DNA copy number were available for 177 patients and the median value was 1,530 copies/ml.

^b^ Data of serum CRP levels were available for 240 patients and the CRP level > 10 mg/L was used as the cutoff value.

### Baseline BMI

The median BMI was 21.2 (range: 13.5-32.4). According to the WHO classification, 17.9% of patients (133) were underweight, 69.8% of patients (518) were normal weight, 11.6% of patients (86) were overweight, and 0.7% of patients (5) were obese. We performed ROC curve analysis to determine the optimal BMI cutoff with which to distinguish between the two groups and found that the cutoff was 19.95. Because a BMI = 25 was used as the cutoff value in several previous studies, and a BMI = 20 was used as the cutoff value in another study [7, 9, 10, 22], we evaluated the prognostic value of each of these BMI cutoff points, as well as the prognostic value of our median BMI (21.2). A BMI ≥ 20 was found to be the threshold value with the smallest P value (P = 0.001 for BMI ≥ 20, P = 0.005 for BMI ≥ 21.2, P = 0.019 for BMI ≥ 25) and was thus considered the most discriminatory threshold value. Based on the results of our ROC analysis, we used a BMI ≥ 20 as the cutoff value in the present study. We defined patients with BMIs < 20 as having low BMIs and patients with BMIs ≥ 20 as having high BMIs. Based on this classification, 254 patients (34.2%) were categorized into the low BMI group, and 488 patients (65.8%) were categorized into the high BMI group. The baseline clinical features of the patients in the low BMI group were compared with those of the patients in the high BMI group (Table 1).

Compared with the high BMI group, the low BMI group featured patients with higher ECOG PS scores (≥ 2), more frequent B symptoms, lower albumin levels, and higher KPI scores. No significant differences regarding other clinical characteristics were observed between the low BMI and high BMI groups (Table 1).

### Treatment modalities and responses

A total of 471 patients (63.5%) received chemotherapy combined with radiotherapy (RT), 179 patients (24.1%) received chemotherapy alone, 74 patients (10.0%) received radiotherapy alone, and 18 patients (2.4%) received only supportive care. Detailed information regarding these treatments and patient responses are listed in Table 2. No significant differences were noted between the low BMI group and high BMI group with respect to treatment modalities (P > 0.05). A total of 540 of the 724 treated patients (74.6%) displayed a complete response (CR) or an unconfirmed complete response (CRu) to their initial treatment. The CR rate was significantly lower in the low BMI group than in the high BMI group (63.4% vs. 77.7%, respectively, P < 0.001).

**Table 2:** Primary treatment and response stratified by BMI (body mass index)

Characteristics	BMI groups		*P*
	Low BMI (< 20)	High BMI (≥ 20)	
Treatment modalities			0.062
CT combined RT	147 (57.9)	324 (66.4)	
CT alone	73 (28.7)	106 (21.7)	
RT alone	25 (9.8)	49 (10.0)	
Best supportive care	9 (3.5)	9 (1.8)	
Chemotherapy regimens			0.766
CHOP or CHOP-like	91 (41.2)	160 (37.3)	
EPOCH	37 (16.7)	71 (16.6)	
ATT	12 (5.4)	33 (7.7)	
GEMOX + L-asp	79 (35.7)	160 (37.3)	
SMILE	2 (0.9)	5 (1.2)	
Mean no. cycles of CT	3.61	3.79	0.240
Complete remission			<0.001
Yes	161 (63.4)	379 (77.7)	
No	93 (36.6)	109 (22.3)	

Abbreviations: CT: chemotherapy; RT: radiotherapy; CHOP: cyclophosphamide + doxorubicin + vincristine + prednisone; EPOCH: etoposide + doxorubicin + vincristine + cyclophosphamide + prednisone; ATT: alternating triple therapy (CHOP-B, cyclophosphamide + doxorubicin + vincristine + bleomycin + prednisone; IMVP-16, ifosfamide + methotrexate + etoposide; DHAP, dexamethasone + cisplatin + cytarabine); GEMOX + L-asp: gemcitabine + oxaliplatin + L-asparaginase; SMILE: dexamethasone + methotrexate + ifosfamide + L-asparaginase + etoposide.

To exclude the potential impact of inadequate chemotherapy on survival among patients with low BMIs, we compared the mean numbers of chemotherapy cycles between the two BMI groups. Among patients receiving chemotherapy (n = 650), no significant difference was noted in the mean number of chemotherapy cycles between the low BMI and high BMI groups (P = 0.240, Table 2). Among patients receiving L-asparaginase-containing chemotherapy (n = 246 cases), no significant difference was noted in the mean number of chemotherapy cycles between the low BMI (mean no. cycles = 4.11, range 1-8) and high BMI groups (mean no. cycles = 3.67, range 1-8) (P = 0.054). Similarly, among patients receiving anthracycline-containing chemotherapy (n = 404 cases), no significant difference was noted in the mean number of chemotherapy cycles between the low BMI (mean no. cycles = 3.42, range 1-8) and high BMI groups (mean no. cycles = 3.80, range 1-9) (P = 0.067). These findings are important because reductions in the numbers of planned chemotherapy cycles necessitated by poor tolerance in low BMI patients may result in inferior survival among these patients.

### Survival analysis stratified by BMI

**Figure 1:**
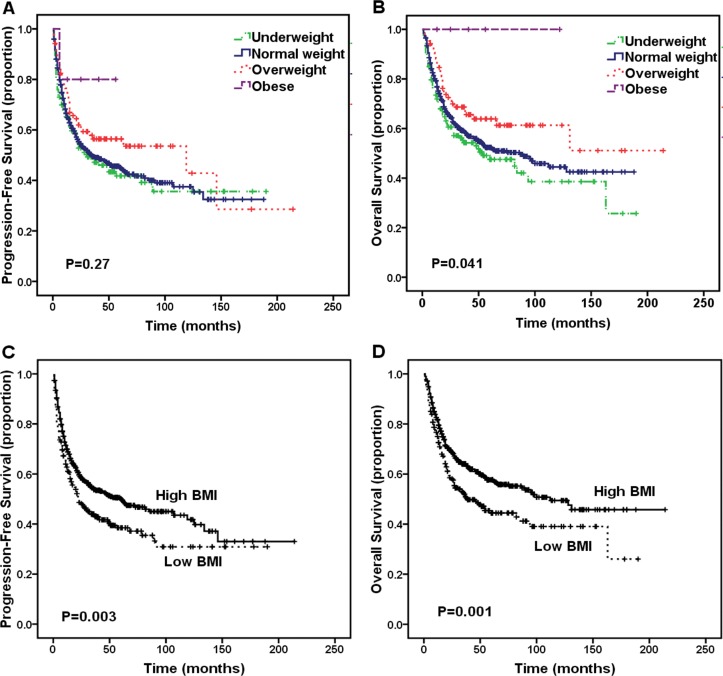
Survival outcome of patients according to different body mass index (BMI) classification A. Progression-free survival (PFS) of patients with extranodal natural killer (NK)/T-cell lymphoma (ENKTL) according to underweight, normal weight, overweight, and obese. B. Overall survival (OS) of patients with ENKTL according to underweight, normal weight, overweight, and obese. C. PFS of patients with ENKTL according to low BMI group versus high BMI group. D. OS of patients with ENKTL according to low BMI group versus high BMI group.

A total of 327 deaths (44.1%) occurred during a median follow-up period of 40 months (range, 1-214 months), and all but 14 of these deaths were caused by tumor progression. The estimated 5-year PFS and OS rates for all 742 patients were 46.1% and 53.1%, respectively. The 5-year PFS rates for underweight, normal weight, overweight and obese patients were 41.8%, 45.1%, 56.4% and 80.0% (P = 0.270, Figure 1A), respectively. The 5-year OS rates for underweight, normal weight, overweight and obese patients were 47.5%, 52.4%, 63.9% and 100%, respectively (P = 0.041, Figure 1B). When the entire cohort was divided into low BMI ( < 20) and high BMI (≥ 20) subgroups, patients in the high BMI group exhibited significantly better PFS (5-year PFS, 50.1% vs. 38.5%, respectively; P = 0.003, Figure 1C) and OS (5-year OS, 57.7% vs. 44.5%, respectively; P = 0.001, Figure 1D) than patients in the low BMI group. Furthermore, patients receiving asparaginase-containing regimens exhibited significantly better PFS (5-year PFS: 64.0% vs. 38.2%, P < 0.001) and OS (5-year OS: 67.7% vs. 48.7%, P < 0.001) than patients receiving anthracycline-containing chemotherapy.

High BMI group exhibited significantly better OS than the low BMI group (P = 0.007) among male patients. High BMI group exhibited better OS, with borderline significance, than the low BMI group (P = 0.076) among female patients. Among patients ≤ 60 years, low BMI was significantly associated with inferior OS (P < 0.001). However, there was no significant difference in OS between the low- and high BMI groups among patients > 60 years (P = 0.984). Low BMI was significantly associated with inferior OS in patients with Ann Arbor stage I/II disease, but not in patients with advanced disease (P = 0.005 and P = 0.17, respectively). Low BMI was also significantly associated with inferior OS (P = 0.004) among patients receiving chemotherapy ± radiotherapy (650 patients, 87.2%), while low BMI was not significantly associated with survival among patients receiving radiotherapy alone (74 cases, 10.0%) (P = 0.382). Among patients receiving L-asparaginase-containing chemotherapy (246 patients, 33.2%), low BMI was significantly associated with inferior OS (P = 0.01). While among patients receiving anthracycline-containing chemotherapy (404 cases, 54.4%), high BMI tended to be associated with better OS than low BMI, but the difference was not statistically significant (P = 0.082).

Table 3 displays the results of the univariate and multivariate analysis of the potential predictors of PFS and OS. Multivariate analysis identified masses > 5 cm (RR = 1.559, 95% CI: 1.153-2.11, P = 0.004), albumin levels < 35 g/L (RR = 1.308, 95% CI: 1.037-1.649, P = 0.023), KPI scores ≥ 2 (RR = 1.337, 95% CI: 1.054-1.696, P = 0.017), IPI scores ≥ 2 (RR = 1.385, 95% CI: 1.141-1.78, P = 0.01) and low BMIs (RR = 1.244, 95% CI: 1.008-1.534, P = 0.041) as adverse predictors of PFS. Multivariate analysis identified age > 60 years (RR = 1.819, 95% CI: 1.383-2.393, P < 0.001), masses > 5 cm (RR = 1.787, 95% CI: 1.303-2.452, P < 0.001), stage III/IV disease (RR = 2.075, 95% CI: 1.549-2.78, P < 0.001), elevated LDH levels (RR = 1.359, 95% CI: 1.069-1.726, P = 0.012), albumin levels < 35 g/L (RR = 1.64, 95% CI: 1.284-2.094, P < 0.001) and low BMIs (RR = 1.331, 95% CI: 1.06-1.67, P = 0.014) as significant independent predictors of OS.

**Table 3:** Univariate and multivariate analysis of prognostic factors for PFS and OS

Factors	PFS			OS		
	Univariate analysis	Multivariate analysis		Univariate analysis	Multivariate analysis	
	*P*	RR (95% CI)	*P*	*P*	RR (95% CI)	*P*
Age > 60 years	0.001			<0.001	1.819 (1.383-2.393)	<0.001
B symptoms	0.052			0.015		
Mass ≥5 cm	<0.001	1.559 (1.153-2.11)	0.004	<0.001	1.787 (1.303-2.452)	<0.001
Extranodal sites ≥2	<0.001			<0.001		
Regional LN involvement	<0.001			0.008		
Stage III/IV	<0.001			<0.001	2.075 (1.549-2.78)	<0.001
LDH >245 U/l	<0.001			<0.001	1.359 (1.069-1.726)	0.012
Low BMI (< 20)	0.003	1.244 (1.008-1.534)	0.041	0.001	1.331 (1.06-1.67)	0.014
Albumin < 35 g/L	<0.001	1.308 (1.037-1.649)	0.023	<0.001	1.64 (1.284-2.094)	<0.001
IPI score ≥2	<0.001	1.385 (1.141-1.78)	0.01	<0.001		
KPI score ≥2	<0.001	1.337 (1.054-1.696)	0.017	<0.001		

Abbreviations: PFS: progression-free survival; OS: overall survival; RR: relative risk; CI: confidence interval; LN: lymph node; LDH: lactate dehydrogenase; BMI: body mass index; IPI: International Prognostic Index; KPI: Korean Prognostic Index.

**Figure 2:**
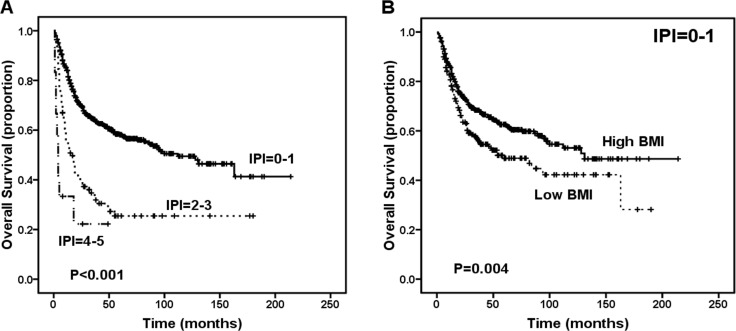
Survival outcome of patients according to the International Prognostic Index (IPI) score A. Overall survival (OS) according to the IPI for patients with extranodal natural killer (NK)/T-cell lymphoma (ENKTL). B. OS of patients with IPI score = 0-1 according to low body mass index (BMI) group versus high BMI group.

Using the IPI predictive model, we determined that 633 patients (85.3%) were in the low-risk group (IPI = 0-1), 97 patients (13.1%) were in the intermediate risk group (IPI = 2-3), and 12 patients (1.6%) were in the high-risk group (IPI = 4-5) with respect to survival outcomes. The 5-year OS rates were 58.1% for the low-risk group, 25.4% for the intermediate-risk group, and 22.2% for the high-risk group (P < 0.001, Figure 2A). Significant differences in survival were also found between the low-risk and intermediate-risk groups (P < 0.001) and between the intermediate-risk and high-risk groups (P = 0.029). However, based on the IPI data, 85.3% of patients were disproportionately grouped into the low-risk group. Additionally, the IPI score was unable to identify patients with different survival statuses within the low-risk group, while the BMI classification (low BMI vs. high BMI) efficiently categorized patients in the low-risk IPI group into two subgroups with different survival outcomes (P = 0.004, Figure 2B).

**Figure 3:**
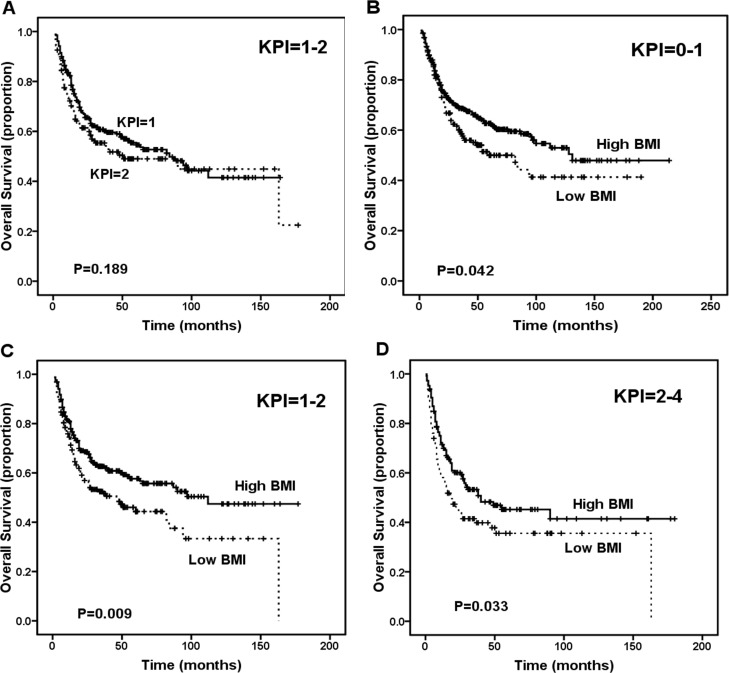
Survival outcome of patients according to the Korean Prognostic Index (KPI) score A. Overall survival (OS) of patients with KPI score = 1-2 according to the KPI model. B. OS of patients with KPI score = 0-1 according to low body mass index (BMI) group versus high BMI group. C. OS of patients with KPI score = 1-2 according to low BMI group versus high BMI group. D. OS of patients with KPI score = 2-4 according to low BMI group versus high BMI group.

The KPI model balanced the numbers of patients in the different risk categories more efficiently than the IPI model (score 0: 243 patients, 32.7%; score 1: 257 patients, 34.6%; score 2: 161 patients, 21.7%; and score 3-4: 81 patients, 10.9%) and was able to distinguish between patients with different survival outcomes. The 5-year OS rates were 63.0% for the KPI = 0 group, 54.6% for the KPI = 1 group, 49% for the KPI = 2 group and 27.6% for the KPI = 3-4 group (P < 0.001). Moreover, the KPI model significantly distinguished between low- and intermediate-low-risk patients (KPI = 0 vs. KPI = 1, P = 0.011) and high-intermediate- and high-risk patients (KPI = 2 vs. KPI = 3-4, P = 0.002), but not intermediate-low- and high-intermediate-risk patients (KPI = 1 vs. KPI = 2, P = 0.189, Figure 3A). In contrast, the BMI classification (low BMI vs. high BMI) was efficient at distinguishing among patients with KPI scores = 0-1 (P = 0.042, Figure 3B), patients with KPI scores = 1-2 (P = 0.009, Figure 3C) and patients with KPI scores = 2-4 (P = 0.033, Figure 3D).

### Survival analysis stratified by the new PINK and PINK-E prognostic models

**Figure 4:**
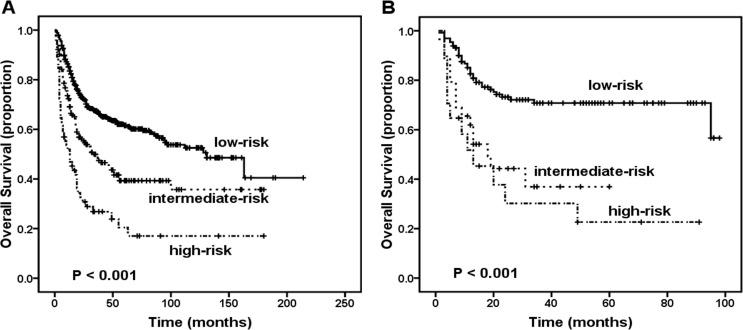
Survival outcome of patients according to the prognostic index for natural killer lymphoma (PINK) and prognostic index for natural killer lymphoma-Epstein-Barr (PINK-E) A. Overall survival by PINK risk group. B. Overall survival by PINK-E risk group.

Kim et al. recently proposed the following two new prognostic models: the prognostic index of natural killer lymphoma (PINK) and the PINK plus Epstein-Barr virus DNA (PINK-E) for patients with ENKTL received non-anthracycline-based treatment [23]. The PINK identified 3 risk groups (low risk: no risk factors, intermediate-risk: one risk factor, and high-risk: two or more risk factors) with different survival outcomes based on the presence or absence of 4 prognostic factors (age greater than 60 years, stage III or IV disease, distant lymph-node involvement, and non-nasal type disease). The PINK-E, which included PINK and EBV DNA, also identified 3 risk groups (low risk: zero risk factors or one risk factor, intermediate-risk: two risk factors, and high-risk: three or more risk factors) with different survival outcomes. Regarding the present population, according to the PINK, we identified three categories of patients with different 5-year OS. The 5-year OS rates were 61.6% for the low-risk group, 39.3% for the intermediate-risk group, and 17.0% for the high-risk group (Figure 4A, P < 0.001), respectively. Similarly, according to the PINK-E, our patients were stratified into three groups with different 5-year OS. The 5-year OS rates were 70.8% for the low-risk group, 36.9% for the intermediate-risk group, and 22.6% for the high-risk group, respectively (Figure 4B, P < 0.001).

### Sensitivity analysis

Data on B symptom (weight loss) were missing for 2 cases in the present cohort. Significant weight loss (weight loss ≥ 10%) was reported in 196 patients (26.5%) in the present study. Sensitivity analyses excluding those patients with significant weight loss, indicating that BMI categories (low- vs. high-BMI) remained statistically significant, with P = 0.006 and 0.001 for PFS and OS among patients without weight loss (n = 544, 73.5%), respectively.

## DISCUSSION

In this study, we found that increased BMI was significantly associated with improved survival outcomes among patients with ENKTL. The association remained significant after adjusting for important baseline prognostic factors or limiting our analysis to patients receiving L-asparaginase-containing chemotherapy. Although the role of BMI in lymphoma prognosis has been intensely studied across different subtypes of the disease, the associations between BMI and ENKTL treatment outcomes remain largely unknown. To the best of our knowledge, this is the first study to assess the prognostic value of BMI in T/NK-cell NHL.

The results of several previous studies examining the impact of BMI on lymphoma patient outcomes were inconsistent, as some studies found that overweight/obesity was associated with worse outcomes [11], while some found no association between overweight/obesity and worse outcomes [13, 24], and others found that it was associated with improved outcomes [6–10]. Hwang et al. found that being underweight or severely obese has a deleterious prognostic impact in DLBCL [25]. Consistent with the findings of several previous studies [6–10], we found that higher BMI is an independent prognostic factor for longer PFS and OS. However, because no studies published in English to date have investigated the prognostic role of BMI in T/NK-cell NHL outcomes, we could not compare our results with published results.

No definitive BMI cutoff values that can be used to predict cancer patient outcomes have been identified. Most authors have used the WHO classification of obesity to distinguish between BMI groups [7, 13, 25, 26]. In contrast, Park et al. [9] and Weiss et al. [10] used dichotomized classification schemes with cutoffs of 20.0 and 25.0 kg/m^2^, respectively. In the present study, based on the WHO classification, the total cohort was split into four groups of patients with different OS rates. However, the distribution of patients was seriously imbalanced, as only 12.3% of patients were categorized into the overweight/obese group, and almost 70% patients were categorized into the normal weight group. After comparing the prognostic value of the abovementioned BMI cutoffs, we determined that a BMI ≥ 20 was the most discriminatory threshold value and that it was also very similar to the optimal cutoff value identified via ROC analysis. Therefore, we adopted BMI ≥ 20 as the cutoff value in the present study.

We have learned several lessons from the present study. It may be worthwhile to investigate the mechanisms underlying the significant differences in outcomes between low BMI and high BMI ENKTL patients. We hypothesize that these differences may be attributable to several phenomena. Previous studies have found that doxorubicin clearance was reduced in obese patients [27, 28], implying that pharmacokinetic or pharmacodynamic differences between obese and non-obese patients may result in a higher physiologic chemotherapy doses in obese patients. However, in the present study, when survival analysis was restricted to patients receiving anthracycline-containing chemotherapy, the BMI classification failed to demonstrate significant associations between BMI and PFS and OS. In contrast, low BMI had an adverse impact on the treatment outcomes of patients receiving L-asparaginase-containing chemotherapy. There was some potential reason why BMI predicts outcome for L-asparaginase-containing regimens and not others. First, several studies have demonstrated that the ENKTL is generally resistant to anthracyclines but generally sensitive to asparaginase which is widely considered an effective treatment option for ENKTL [17, 29–31]. We speculate that the generally resistance of ENKTL leads to the loss of predictive value of BMI for anthracyclines-containing regimens. Second, we found that low BMI was associated with hypoalbuminemia which may have an important effect on the pharmacokinetics and clearance of chemotherapeutic drugs. Asparaginase can often cause a decrease of serum albumin levels. However, the anthracyclines-containing regimens have little effect on the serum albumin concentration. These may partly explain why body mass index predicts outcome for asparaginase-containing regimens but not anthracyclines-containing regimens. We hypothesized that high BMI patients are exposed to higher cumulative chemotherapy (e.g., doxorubicin, asparaginase and gemcitabine) doses or experience longer periods of drug exposure, resulting in better outcomes. We also hypothesized that low BMI patients may have poor physical stamina, weak immunity due to malnutrition and an increased risk of comorbidities. These patients are also at risk for overdose and chemotherapy-related toxicity [32]. The present study found that patients in the low BMI group were more likely to have hypoalbuminemia, which is the most widely used maker of malnourishment.

These results may have far-reaching consequences with respect to the interpretation and design of clinical trials in the asparaginase era. The 15.2% difference in 5-year overall survival (data not shown) between low BMI and high BMI ENKTL patients who received L-asparaginase-containing chemotherapy reported herein is comparable with those reported in other studies in which asparaginase-containing regimens were used [31]. Depending on their pharmacokinetics, two equipotent drugs or regimens tested in a clinical trial may be associated with significantly different outcomes if groups are unbalanced with respect to BMI. Therefore, we advocate using BMI along with other parameters to ensure appropriate patient stratification, although doing so may increase the complexity of patient stratification.

The present study had several important strengths. First, the cohort was large and comprised patients with a single lymphoma subtype, and the follow-up was both long and comprehensive. Second, because all patients were hospitalized, we were able to collect detailed and important clinical data and calculate BMIs from measured heights and weights rather than self-reported values. Third, large numbers of patients were treated with different regimens at three different centers and were thus managed using a variety of practice patterns, which enhanced the generalizability of our results.

Several potential limitations should also be noted. First, the study was retrospective, and we were unable to examine the effects of several possible confounding factors, such as physical activity or weight gain/loss, during either treatment or follow-up. Second, therapy-related heterogeneity may have confounded the results. Finally, it is conceivable that residual confounding was caused by the presence of unknown or unmeasured variables, such as treatment dose intensities, dose densities and toxicities.

## CONCLUSIONS

In conclusion, we found that low BMI at the time of diagnosis is an independent adverse prognostic factor in ENKTL patients. Using the BMI classification may improve the IPI and KPI prognostic models.

## MATERIALS AND METHODS

### Ethics statement

Written informed consent to draw patient blood samples and to store other medical information in our hospital database was obtained from all patients, who also provided consent to participate in this study. This study was approved by the Institutional Review Board of the National Cancer Institute and the ethics committees of Sun Yat-Sen University Cancer Center, Hunan Cancer Hospital and the Second Xiangya Hospital of Central South University. This study was performed in accordance with the Declaration of Helsinki and the institutional guidelines of the abovementioned local ethics committees.

### Patient selection

We performed a triple-center retrospective study of 742 consecutive patients with newly diagnosed ENKTL, nasal type, at the following three cancer centers: Sun Yat-Sen University Cancer Center, Hunan Cancer Hospital and the Second Xiangya Hospital of Central South University between January 1998 and June 2015. The following patients were included in this study: (a) patients with a pathologically confirmed diagnosis of ENKTL, nasal type, according to the WHO classification [33]; (b) patients without a previous history of malignancy or anti-cancer treatment or a second primary tumor; (c) patients with available height and weight data at the time of diagnosis; and (d) patients with adequate clinical, laboratory, and follow-up data. Patients with blastic NK-cell lymphoma/leukemia, aggressive NK-cell lymphoma/leukemia, or peripheral T-cell lymphoma, unspecified, were excluded.

All pathological specimens were reviewed and reclassified via central review, in accordance with the WHO criteria for pathological diagnosis. Antibodies to the following antigens were used for immunophenotype analysis: CD3, CD56, TIA-1, Gram-B, CD45RO, CD20, CD79a, CD30, Ki67, and anaplastic large cell lymphoma kinase. In situ hybridization was used to detect EBV-encoded RNA.

Before treatment, the following baseline clinical data were collected: patient demographic information, heights, weights, Eastern Cooperative Oncology Group performance statuses (ECOG PSs), B symptoms, treatment modalities and responses, serum lactate dehydrogenase (LDH) levels, baseline serum C-reactive protein (CRP) levels, albumin levels, plasma Epstein-Barr virus-DNA (EBV-DNA) copy numbers, and Ann Arbor stages. Additionally, patients underwent computed tomography (CT) or magnetic resonance (MR) imaging of the nasopharynx, neck, chest, abdomen, and pelvis or positron emission tomography/computed tomography (PET/CT) of the entire body. All patients were staged using the Ann Arbor staging system. The IPI (age, ECOG PS, stage, LDH level, extranodal site involvement) and KPI for nasal NK/T-cell lymphoma (stage, LDH level, B symptoms, regional lymphoma node involvement) were also used to perform survival analyses [34].

### Measurements and definitions

BMI was calculated as weight measured in kilograms divided by the square of height measured in meters (kg/m^2^). Consistently recorded height data from any clinical history time point were considered accurate. Weight at diagnosis was defined as the weight measured closest to and no earlier than 1 month before the ENKTL diagnosis date. Patients were stratified according to the World Health Organization (WHO) international BMI classification as follows: underweight (BMI < 18.5), normal weight (BMI ≥ 18.5 to < 25), overweight (BMI ≥ 25 to < 30), and obese (BMI ≥ 30). Patients were also stratified according to the indicated 2-sided (BMI < or ≥ 20) classification.

### EBV-DNA quantification

Patient plasma samples were collected within one week before treatment initiation. Approximately 4-5 mL venous blood was collected into tubes containing EDTA anticoagulant, incubated at 4°C for 30 minutes, centrifuged for 10 minutes at 1485g, 1.5-2 mL plasma was collected and frozen at −80°C before further processing. Total plasma cell-free DNA was isolated using a QIAamp Blood Mini Kit (QIAGEN, Inc., Valencia, CA, USA) and eluted in 100-μL sterile, deionized-distilled water. Finally, 2-μL aliquots were used for the quantitative RT-PCR assay. The BamHI-W assay was performed as previously described to generate an EBV DNA-containing plasmid [35]. The primer and probe sequences were as follows: forward, 5′-CCCAACACTCCACCACACC-3′; reverse, 5′-TCTTAGGAGCTGTCCGAGGG- 3′; probe, 5′-FAM-CACACACTACACACACCCACCCGTCTC-TAMRA-3′ (Invitro -gen). Each 25-μL PCR reaction contained 1 μL plasma DNA template, 2.5 μL Premix Gold buffer containing deoxynucleotide triphosphates (Takara), 10 pmol forward primer, 10 pmol reverse primer, 0.5 U TaqGold (Applied Biosystems), and deionized H_2_O. The PCR assay was performed at 93°C for 5 minutes, followed by 40 cycles of 95°C for 30 seconds, 55°C for 30 seconds, and 72°C for 30 seconds with a final extension of 72°C for 10 minutes. The PCR product was cloned into the pGM-T vector (Tiangen Biotech), purified using the QIAamp Blood Mini Kit (QIAGEN), and confirmed by sequencing using the ABI 3100 DNA Sequence Detector (Applied Biosystems).

Each 96-well plate included triplicate samples run in parallel with the EBV-DNA plasmid standard curve dilutions. The BamHI-W standard curve spanned 5-logs comprising serial 10-fold dilutions, containing 10^0^-10^4^ copies of EBV-DNA plasmid target per well. EBV-negative healthy volunteers were used as negative controls, and a no template control was run on each plate as a blank control.

The 25-μL real-time quantitative RT-PCR reaction mixture contained 2μL EBV DNA plasmid or plasma DNA sample, 12.5 μL Premix Gold buffer containing deoxynucleotide triphosphates, 5 pmol forward primer, 5 pmol reverse primer, and 2.5 pmol of the probe, as previously described, in addition to 1.5 U TaqGold and deionized H_2_O. The quantitative RT-PCR assay was performed over 45 cycles of 93°C for 3 minutes, 93°C for 30 seconds, and 55°C for 45 seconds on the ABI 7500 Fast Real-Time PCR System (Applied Biosystems), according to the manufacturer's instructions. Fluorescence data were collected in real-time and analyzed using the Sequence Detection System Version 1.9 software. The results were expressed as the number of copies of EBV per milliliter of plasma. Samples showing fluorescence signals lower than the signal in the 10^1^ standard were outside the linear range of the assay and considered to be negative.

### Response criteria and statistical analysis

Treatment response was assessed according to the International Working Group Recommendations for Response Criteria for non-Hodgkin Lymphoma [36]. Progression-free survival (PFS) was defined as the interval between the date of diagnosis and the date of first relapse, progression, or death from any cause or the last date on which patients were censored. Overall survival (OS) was defined as the interval from the date of diagnosis until either the date of death from any cause or the last date on which patients were censored. Receiver-operating-characteristic (ROC) analysis was used to determine an optimal BMI cutoff value for predicting disease progression or death. The relationships between BMI and patient clinical and laboratory variables were assessed using Pearson's chi-square test or Fisher's test for categorical variables. The log-rank test and Kaplan-Meier method were applied for univariate survival analysis. Variables significant at P < 0.05 in the univariate analysis were included in the multivariate analysis. Multivariate analysis was performed according to the Cox proportional hazards model. A two-tailed P-value < 0.05 was considered statistically significant. The statistical software package SPSS 16.0 (SPSS, USA) was used for statistical calculations.
